# Effect of norepinephrine dosage on mortality in patients with septic shock

**DOI:** 10.1186/s40560-018-0280-1

**Published:** 2018-02-26

**Authors:** Hitoshi Yamamura, Yu Kawazoe, Kyohei Miyamoto, Tomonori Yamamoto, Yoshinori Ohta, Takeshi Morimoto

**Affiliations:** 1 Department of Disaster and Critical Care Medicine, Hirosaki University School of Medicine, 5 Zaifuchou, Hirosaki, Aomori 036-8562 Japan; 2 Division of Emergency and Critical Care Medicine, Tohoku University, Sendai, Japan; 3 Department of Emergency and Critical Care Medicine, Wakayama Medical University, Wakayama, Japan; 4 Department of Trauma and Critical Care Medicine, Osaka City University, Osaka, Japan; 5 Division of General Medicine, Hyogo College of Medicine, Nishinomiya, Japan; 6 Department of Clinical Epidemiology, Hyogo College of Medicine, Nishinomiya, Japan

**Keywords:** Norepinephrine, Septic shock, Ventilator-free days

## Abstract

**Background:**

Use of high-dose norepinephrine is thought to have an immunosuppressive action that increases mortality. This study aimed to evaluate the correlation between norepinephrine dosage and prognosis of patients with septic shock.

**Methods:**

This study was a nested cohort of the DExmedetomidine for Sepsis in Intensive Care Unit Randomized Evaluation (DESIRE) trial. We evaluated 112 patients with septic shock and an initial Sequential Organ Failure Assessment Cardiovascular (SOFA-C) category score > 2 and initial lactate level > 2 mmol/L. We divided the patients into two groups according to the norepinephrine dosage administered over the initial 7 days: high dose (≥ 416 μg/kg/week) (H group, *n* = 56) and low dose (< 416 μg/kg/week) (L group, *n* = 56). The primary outcome of interest was 28-day mortality. Secondary outcomes were ventilator-free days, initial 24-h infusion volume, initial 24- to 48-h infusion volume, and the need for renal replacement therapy. For comparisons between the H group and L group, we used the chi-square test or Fisher’s exact test for categorical variables and the *t* test or Wilcoxon rank sum test for continuous variables. For time-to-event outcomes, Cox proportional hazards models were used. Kaplan-Meier survival curves were created for graphical representation.

**Results:**

Patient characteristics appeared to be similar between the two groups except for the SOFA-C score and fibrinogen degradation product level. The cumulative incidence of death at 28 days was 29.9% (16 patients) in the L group and 29.7% (15 patients) in the H group (*p* = 0.99). The median number of 28-day ventilator-free days was 20 (0, 25) in the L group and 16 (0, 22) in the H group (*p* < 0.05). Initial infusion volume at 0–24 h in the H group was significantly higher than that in the L group (*p* = 0.004). Infusion volume at 24–48 h in the H group was also significantly higher than that in the L group (*p* = 0.03).

**Conclusions:**

No statistically significant difference was observed in 28-day mortality between patients with septic shock treated with high-dose norepinephrine compared with those treated with low-dose norepinephrine. However, the number of ventilator-free days in the L group was higher than that in the H group.

**Trial registration:**

clinicaltrials.gov Identifier: NCT01760967 Date of trial registration: January 4, 2013.

## Background

Norepinephrine is the vasopressor of first choice for patients with septic shock [1]. Norepinephrine recruits unstressed volume through alpha adrenergic effects on venous and arterial vessels and might recruit volume to the macrovasculature. However, norepinephrine is also thought to have an immunosuppressive action that causes a poor prognosis [2, 3]. Previous reports showed that norepinephrine dosage was associated with intensive care unit (ICU) mortality, with an especially high mortality rate at doses above 1 μg/kg per min [2]. From this previous study, the high-dose usage of norepinephrine was thought to cause high mortality in patients with sepsis. As another problem, in the treatment strategy of septic shock, it is important to include early recognition, fluid resuscitation, and maintenance of the blood pressure. However, if massive fluid resuscitation is required, this can cause pulmonary edema and prolonged the number of ventilator days. In this study, we aimed to evaluate the correlation between norepinephrine dosage and prognosis and the number of ventilator-free days (VFD) of patients with septic shock.

## Methods

### Patient selection

**Fig. 1:**
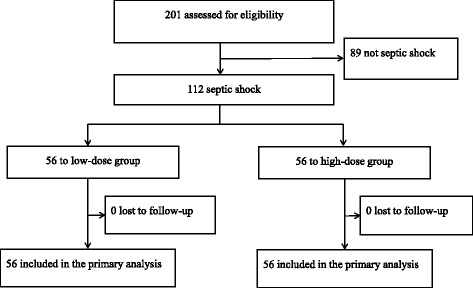
Flow of participants in the norepinephrine dosage for septic shock study

The DExmedetomidine for Sepsis in Intensive Care Unit Randomized Evaluation (DESIRE) trial was conducted from February 2013 to January 2016 [4]. This trial was a multicenter, randomized, controlled trial that enrolled 201 adult patients with sepsis undergoing ventilation. It was designed to assess the effects of a sedation strategy with dexmedetomidine compared with that without dexmedetomidine. The results of this trial in the 201 patients showed that treatment with dexmedetomidine vs that without dexmedetomidine did not significantly reduce the number of VFD (20 vs 18 days) or 28-day mortality (23 vs 31%, hazard ratio 0.69). This sub-analysis of the 201 randomized patients included those with septic shock. Septic shock was defined as a Sequential Organ Failure Assessment (SOFA) score > 2 for the cardiovascular category and a lactate level > 2 mmol/L at randomization. We enrolled 112 patients and divided the patients into two groups according to the total dosage of norepinephrine administered over the initial 7 days: low dose (< 416 μg/kg/week) (L group, *n* = 56) and high dose (≥ 416 μg/kg/week) (H group, *n* = 56) (Fig. 1).

### Treatment protocol

The treatment protocol for sepsis was based on the Guidelines for the Management of Sepsis [1]. In the resuscitation from septic shock-induced hypoperfusion, we initially administered an adequate amount of crystalloid on admission to maintain a mean arterial pressure of 65 mmHg, central venous pressure of 8–12 mmHg, and urinary output of > 0.5 mL/kg/h. Following fluid resuscitation, if the blood pressure could not be maintained, we used norepinephrine or vasopressin as the vasopressor.

### Measurements

We collected data on the initial serum lactate level, SOFA score, and Acute Physiology and Chronic Health Evaluation II (APACHE II) score at randomization. White blood cell (WBC) count, levels of fibrinogen, D-dimer, fibrinogen degradation products (FDP), C-reactive protein (CRP), and procalcitonin (PCT) and norepinephrine dosage were assessed. Infusion volume was assessed on the first and second days, and the dosages of other vasopressors were assessed on the first 7 days after randomization.

The primary outcome of interest was 28-day mortality. For other outcomes, patients were followed in the hospital from enrollment for 28 days or until discharge or death if earlier. Secondary outcomes included the number of VFD, defined as the number of days without use of a ventilator during the 28-day study period, initial 24-h infusion volume, initial 24- to 48-h infusion volume, and the need for renal replacement therapy including continuous renal replacement therapy and hemodialysis.

### Statistical analysis

Continuous variables are presented as the mean ± standard deviation (SD) or the median and interquartile range (IQR). Categorical variables are presented as numbers and percentages (%). For comparisons between the H group and L group, we used the chi-square test or Fisher’s exact test for categorical variables and the *t* test or Wilcoxon rank sum test for continuous variables.

For time-to-event outcomes (time to ICU discharged death), Cox proportional hazards models were used. Kaplan-Meier survival curves were created for graphical representation of these time-to-event outcomes. When examining 28-day mortality, patients were censored at the time of last contact while alive or at 28 days from enrollment, whichever came first. Censoring for hospital discharge analyses occurred at the time of death or, rarely, at study withdrawal. To account for any effect of site and for baseline imbalances, a Cox proportional hazards regression model was used with patients nested within site, and site treated as a random effect with the following covariates included in the model: APACHE II score > 23, age > 65, emergency operation, infection site is lung, and treated with dexmedetomidine. A two-sided *p* value of < 0.05 was considered statistically significant, and all analyses were performed using JMP Pro software (version 12.2; SAS Institute Inc., Cary, NC, USA).

## Results

Patient characteristics appeared to be similar between the two groups except for the Sequential Organ Failure Assessment Cardiovascular (SOFA-C) score and FDP level (Table 1). In the H group, use of another vasopressor, such as dobutamine, and total vasopressin dosage within 7 days were significantly higher than those in the L group. Causes of sepsis were lung (*n* = 29), abdomen (*n* = 52), and others (*n* = 31).

**Table 1:** Patient characteristics

	L group (*n* = 56)	H group (*n* = 56)	*p* value
Age, years	70.8 ± 13.4	70.5 ± 14.4	0.92
Male sex, *n* (%)	33 (58)	36 (64)	0.56
Body weight, kg	53.9 ± 11.2	54.7 ± 11.9	0.72
COPD (%)	4 (7.1)	3 (5.3)	0.70
Soft tissue infection (%)	4 (7.1)	4 (7.1)	1.00
Emergency surgery (%)	28 (50.1)	23 (41.1)	0.34
Site of infection (%)			
Lung	16 (29)	13 (23)	
Abdomen	29 (52)	23 (41)	
Urinary tract	4 (7)	8 (14)	
Skin and soft tissue	1 (2)	6 (11)	
Others	6 (11)	6 (11)	
APACHE II score	25 (19, 33)	25 (20, 30)	0.89
SOFA score	10 (8, 12)	10 (8, 12)	0.63
SOFA-R score	2 (1, 3)	2 (1, 3)	0.65
SOFA-P score	0.5 (0, 2)	1 (0, 2)	0.23
SOFA-L score	0 (0, 1)	0 (0, 1)	0.65
SOFA-C score	3 (3, 4)	4 (3, 4)	0.007
SOFA-N score	0 (0, 3)	1 (0, 2)	0.63
SOFA-K score	1.5 (0, 3)	1 (0, 2)	0.34
Systolic BP, mmHg	109 (26)	105 (28)	0.31
Mean BP, mmHg	73 (16)	72 (18)	0.75
Lactate level, mmol/L	4.5 (3.0, 7.8)	4.4 (3.6, 6.6)	0.94
Urine output, mL/day	1240 (298, 2302)	1279 (378, 2566)	0.84
WBC, mm^3^	8500 (4500, 14,109)	5000 (2250, 13,930)	0.18
FDP, μg/dL	15.8 (7.5, 28.0)	23.6 (10.5, 52)	0.02
Fibrinogen, mg/dL	337 (243, 532)	403 (271, 583)	0.26
CRP, mg/dL	11.9 (5.2, 24.4)	16.1 (5.4, 27.3)	0.76
PCT, ng/mL	29.3 (3.2, 81.5)	40.0 (12.9, 100)	0.11
Catecholamine			
Total dopamine dosage (μg/kg)	15,727 (6180, 36,150)	28,532 (12,321, 43,407)	0.15
Total dobutamine dosage (μg/kg)	6191 (3652, 14,796)	23,051 (13,931, 35,760)	0.003
Total vasopressin dosage (IU)	9.8 (5.1, 15.4)	30.2 (12, 54.2)	0.05
Hospital length of stays, days	29 (31)	33 (29)	0.12
Renal replacement therapy (%)	18 (32)	32 (57)	0.008

Data are shown as mean ± SD, number of subjects (%), or median (IQR), as appropriate

*SD* standard deviation, *COPD* chronic obstructive pulmonary disease, *IQR* interquartile range, *APACHE II* Acute Physiology and Chronic Health Evaluation II, *SOFA* Sequential Organ Failure Assessment, *SOFA-R* Sequential Organ Failure Assessment Respiration score, *SOFA-P* Sequential Organ Failure Assessment Coagulation score, *SOFA-L* Sequential Organ Failure Assessment Liver score, *SOFA-C* Sequential Organ Failure Assessment Cardiovascular score, *SOFA-N* Sequential Organ Failure Assessment Central nervous system score, *SOFA-K* Sequential Organ Failure Assessment Renal score, *BP* blood pressure, *WBC* white blood cell, *FDP* fibrinogen degradation products, *CRP* C-reactive protein, *PCT* procalcitonin

**Fig. 2:**
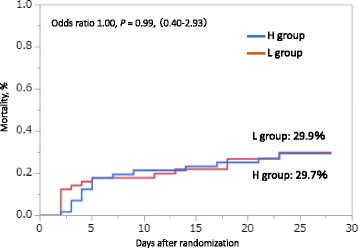
Twenty-eight-day mortality between the high-dose group and low-dose group

**Fig. 3:**
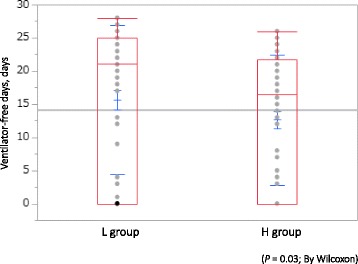
Ventilator-free days between the high-dose group and low-dose group. *p* = 0.03; by Wilcoxon

**Fig. 4:**
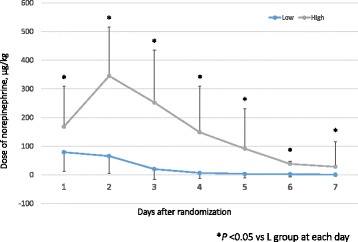
Dose of norepinephrine on each day. *p < 0.05 vs L group at each day

As the primary outcome, the cumulative incidence of death at 28 days was not significantly different between the two groups: 29.9% (16 patients) in the L group and 29.7% (15 patients) in the H group (*p* = 0.99) (Fig. 2). The analysis adjusted for infusion volume over the first 24 h also did not show a significant difference (*p* = 0.38). The median 28-day VFD in the L group was significantly higher than that in the H group (20 [0, 25] vs 16 [0, 20] days: *p* < 0.05) (Fig. 3). Using the Cox proportional hazards model to adjust for all five of the covariates, VFD was incorporated into the model, with similar results compared with the primary analysis. The dose of norepinephrine used was significantly different between the two groups on each of the first 7 days. Especially, the highest dose of norepinephrine administered was in the H group on day 2 at 345.1 (170.9) μg/kg (Fig. 4).

Initial infusion volume at 0–24 h in the H group was significantly higher than that in the L group (7829 [5689, 10,676] vs 5544 [3985, 8000] mL, *p* = 0.004). Infusion volume at 24–48 h in the H group was also significantly higher than that in the L group (3530 [2382, 4612] vs 2689 [1962, 3916] mL, *p* = 0.03). Within the first 3 days after admission, 7 patients died in the H group and 9 patients died in the L group. The cumulative incidences of death at 28 days except for the patients with death within 3 days were not significantly different between the two groups: 32.8% in the L group and 28.4% in the H group (*p* = 0.39). Renal replacement therapy was performed in 32 patients in the H group and in 18 patients in the L group.

## Discussion

Septic shock is defined as a subset of sepsis in which underlying abnormalities of circulatory and cellular metabolism are profound enough to substantially increase mortality [5]. Norepinephrine is the vasoactive agent of first choice for patients with septic shock after adequate volume resuscitation [1]. Our results showed that the dosage of norepinephrine did not affect the mortality of patients with septic shock, but the number of VFD was lower in the H group. The reason for the difference in the number of VFD between the two groups was that the infusion volume in the H group was significantly higher than that in the L group. Massive infusion volumes can bring about pulmonary dysfunction and cardiovascular failure. Generally, such conditions require ventilator support. Thus, we thought that the factors contributing to the lower number of VFD in the H group were the unstable circulatory status and massive infusion volume administered. A previous report showed that a norepinephrine dosage of 1 μg/kg per minute was associated with an ICU death rate of 90% and suggested that a dosage of norepinephrine greater than 1 μg/kg per minute is an independent factor associated with mortality in patients with septic shock [2]. However, the study by Martin and colleagues had a few problems related to fluid treatment for septic shock. The non-survivors group did not receive the same resuscitation infusion volume as the survivors group. Crystalloid was 1.0 L (0.0–2.5) in the 168 survivors vs 1.0 L (0.0–2.0) in the 156 non-survivors, and cumulative fluid administration was 1.5 L (0.9–3.0) in the 168 survivors vs 1.0 L (0.5–2.0) in the 156 non-survivors [2]. These results indicate that the non-survivors were not infused with an adequate amount of resuscitation volume in the initial period.

In our study, the H group received an adequate amount of resuscitation fluid compared with the L group over the initial 24 h and at 48 h. The most important treatment strategy for patients with septic shock is initial fluid resuscitation and maintenance of the blood pressure. If patients with septic shock receive adequate infusion of fluid volume, the dose of norepinephrine may not be related to patient prognosis.

In previous in vitro and animal studies, norepinephrine was shown to exert multiple anti-inflammatory actions [6, 7]. Exogenous norepinephrine infused into the portal vein of rats resulted in elevation of serum levels of IL-10 and IL-1 beta [8, 9]. Another study showed neutrophils incubated with norepinephrine displayed an immunosuppressive phenotype [10–12]. These studies indicate that epinephrine may have anti-inflammatory effects. In contrast, clinical studies have not investigated norepinephrine in relation to immunosuppressive reactions. Some studies investigating the correlation of the dosage of norepinephrine with mortality indicated that a high norepinephrine level is associated with high mortality in patients with septic shock [13]. However, no study found any correlation between the dosage of norepinephrine and immunological parameters. The blocking action of endogenous catecholamine with β-blockers has improved the prognosis in patients with sepsis [14, 15] and reduced secondary infection in pediatric burn patients [16]. These clinical studies suggested that a high catecholamine level may have led to immunoparalysis [17, 18].

In our study, some alternative vasopressors were also used to treat the patients with septic shock. More dobutamine, vasopressin, and renal replacement therapy were used in the H group than in the L group. However, mortality was not significantly different between the two groups. Our results indicated that renal replacement therapy and total dobutamine dosage also did not affect mortality. We surmise that because of the greater inflammatory action in the H group, the patients did not respond to the epinephrine effect and required the use of vasopressin and another vasopressor to maintain their blood pressure. The patients in a severe condition died earlier, and as a result, the doses of norepinephrine or another vasopressor in these patients might be smaller. We also assessed the incidence of death at 28 days after excluding the patients who died within 3 days. However, there was no significant difference between the two groups, and thus we thought that the early death of some patients had no influence on mortality.

Several adverse effects of catecholamines were reported previously, such as pulmonary edema, bowel ischemia, immunomodulation, increase cellular energy expenditure, and hyperglycemia [19–21]. Generally, we believed that a high concentration of catecholamine would increase mortality and worsen patient prognosis. However, our results were contrary to those of previous reports and did not indicate that high norepinephrine usage worsened mortality or caused organ dysfunction such as bowel ischemia and pulmonary edema although we did not measure the actual catecholamine concentration in serum. We think that high-dose norepinephrine may be used safely with no associated complications.

This study has several limitations. First, it was a nested cohort of a randomized control study, and use of a vasopressor other than norepinephrine was not allowed by the treatment protocol. Our study concentrated on the use of noradrenaline as the initial vasopressor, and use of another vasopressor was uneven. Second, use of an alternate vasopressor other than norepinephrine was left to each physician’s judgment. Third, we cannot determine to what extent the mechanism of norepinephrine contributed to the change in mortality. Also, the duration of shock was similar because there was no significant difference in initial lactate levels and APACHE II scores between the two groups. However, the initial SOFA-C score was different. We attribute this difference in SOFA-C score to the catecholamine dosage in the two groups because the initial blood pressure was not different between the groups. The early recognition and treatment of septic shock in our patients may be one factor influencing our results. However, the greater inflammatory action occurring in the H group required a high-dose vasopressor.

## Conclusions

There was no statistically significant difference in 28-day mortality between the patients with septic shock treated with high-dose norepinephrine vs those treated with low-dose norepinephrine. However, the number of VFD was significantly higher in the group treated with low-dose norepinephrine than in the group treated with high-dose norepinephrine.

## Abbreviations

APACHE II: Acute Physiology and Chronic Health Evaluation II
CRP: C-reactive protein
FDP: Fibrinogen degradation products
ICU: Intensive care unit
IQR: Interquartile range
PCT: Procalcitonin
SD: Standard deviation
SOFA: Sequential Organ Failure Assessment
SOFA-C: Sequential Organ Failure Assessment Cardiovascular
VFD: Ventilator-free days
WBC: White blood cell
